# Face-to-Face Versus Mobile Versus Blended Weight Loss Program: Randomized Clinical Trial

**DOI:** 10.2196/mhealth.7713

**Published:** 2018-01-11

**Authors:** Emalie Hurkmans, Christophe Matthys, An Bogaerts, Leonie Scheys, Karlien Devloo, Jan Seghers

**Affiliations:** ^1^ Department of Movement Sciences University of Leuven Leuven Belgium; ^2^ Department of Social Affairs and Health Ecorys Rotterdam Netherlands; ^3^ Department of Endocrinology University Hospitals Leuven Leuven Belgium; ^4^ Department of Chronic Diseases, Metabolism and Ageing University of Leuven Leuven Belgium; ^5^ Department of Clinical and Experimental Endocrinology University of Leuven Leuven Belgium; ^6^ Faculty of Movement and Rehabilitation Sciences University of Leuven Leuven Belgium

**Keywords:** obesity, weight loss, mobile applications, diet

## Abstract

**Background:**

Conventional face-to-face weight loss and weight control programs are very labor intensive for both the patient and the provider. It is unclear to what extent conventional programs can be (partially) completed by mobile health (mHealth) apps.

**Objective:**

The aim of this study was to compare the effectiveness of different weight loss programs using a combination of conventional and mobile programs among adults who are overweight (body mass index [BMI]>29 kg/m²).

**Methods:**

A single-blinded randomized controlled trial among obese adults was performed from September 2015 to March 2016. The study took place in Leuven, Belgium. Of the 102 eligible (BMI >29 kg/m²) adults, 81 (79%) completed the study. The three intervention groups consisted of a conventional face-to-face weight loss program, a weight loss app program (app group), and a partial face-to-face and partial app program (combi group). All intervention groups received the same advice from a dietician and a physical activity coach during a 12-week period. The control group did not receive any information during the same period. Primary outcomes were weight reduction (5% decrease of baseline weight in kg), BMI, metabolic risk factors, dietary pattern, and physical activity.

**Results:**

Significant more participants in all three intervention groups lost at least 5% or more of their weight at baseline compared with the control group. No significant difference was found between the combi group and the conventional group. A trend was found that more participants in the combi group lost 5% or more compared with the app group (19%), *P*=.06. A significant time x group effect was found for BMI and metabolic risk factors, with the control group having the worst results and the combi group being significantly better with regard to BMI compared with the app group. No significant group x time effects were found for the intake of different food and drinks and moderate to vigorous physical activity (MVPA).

**Conclusions:**

The results of this study show that a conventional weight loss program could partially be completed with an mHealth program without affecting the effectiveness.

**Trial Registration:**

Clinicaltrials.gov NCT02595671; https://clinicaltrials.gov/ct2/show/NCT02595671 (Archived by WebCite at http://www.webcitation.org/6w1H0x1Q6)

## Introduction

### Background

Obesity remains a serious global health challenge. Approximately 37% (2.1 billion) of the adult world population is overweight or obese, with a prevalence of over 60% in Australia and the United States and between 15% and 30% in Europe [1]. Overweight and obesity were estimated to cause 3.4 million deaths, 3.9% of years of life lost, and 3.8% of disability adjusted life years, globally [1]. Given the still increasing magnitude of the epidemic and the association between excess weight and cardio-metabolic risk factors and comorbidities including diabetes, certain cancers, heart disease, stroke, thrombotic disease, osteoarthritis, sleep apnea, and liver and pulmonary disease [2], apart from prevention, new treatment interventions that reach a wide population are required to address this major public health problem.

Conventional face-to-face weight loss and weight control programs, including components for healthy eating and physical activity, have been found to be effective [3,4]. Unfortunately, these programs were found to be very labor intensive for both the patient and the health professional because of the frequent, lengthy clinic visits (hour-long visits, often weekly, for several months or longer). This places a high burden on the health care professionals and patients. Furthermore, such programs are only effective when patients are committed to invest over a long time period [3].

### New Developements

New generations of mobile health (mHealth) technologies that make use of mobile phones or tablets for delivering health information and real-time tailored feedback are emerging and offer good potential for delivery of weight loss programs that are less labor intensive [5]. More specific, there is growing interest into the use of mobile apps to deliver weight loss programs because of their low cost advantages and ability to reach a large number of people because of the increasing number of mobile phone ownership [6-8]. So far, multiple freely available mobile weight loss apps are available at the commercial market (eg, Google Play Store and iTunes App Store). A recent review concluded that computer-tailored and mobile interventions positively affect lifestyle behavior up to 1 year [8]. However, when compared with conventional face-to-face interventions, mHealth interventions seem to be less effective [7]. Moreover, the information quality and evidence-based content of mHealth apps needed improvement [4].

So far, most studies evaluated the effectiveness of a conventional face-to-face weight loss program, an mHealth weight loss program, or a conventional program plus mHealth. It remains unclear to what extent a conventional face-to-face weight loss program could (partially) be completed with a weight loss app. Therefore, the aim of this study was to compare the effectiveness of three weight loss programs (eg, conventional face-to-face weight loss program, a mobile weight loss app [app group], and a partial face-to-face or partial app program [combi group]) with no intervention program among adults with obesity (BMI>29 kg/m²).

## Methods

### Participants

From September 2015 to November 2015, overweight adults living in the Leuven (Belgium) region were recruited for this single-blinded randomized controlled trial (RCT). Inclusion criteria included a body mass index (BMI; calculated as weight in kilograms divided by height in meters squared) between 29 and 34 kg/m² (based on patient metabolic characteristics visiting registered dieticians and qualified physical activity coach in a primary care setting), in the age range of 18 to 65 years, having an email address, and having a personal computer or tablet, or mobile phone. The exclusion criteria were suffering from a known physical (eg, orthopedic limitations and stroke) and/or psychological (eg, eating disorders and depression) disease or comorbidity, intake of any medication with possible impact on body weight, endurance capacity, currently treated for diabetes (both type 1 and 2), sleep apnea determined during the last year, a history of systematic strength or endurance training (moderate to high intensity training more than once a week) in the year before the beginning of the trial, a history of following a supervised dietary advice in the year before the beginning of the trial, having a history of bariatric surgery or any other malabsorption-related disease, and pregnancy.

### Recruitment and Randomization

**Figure 1:**
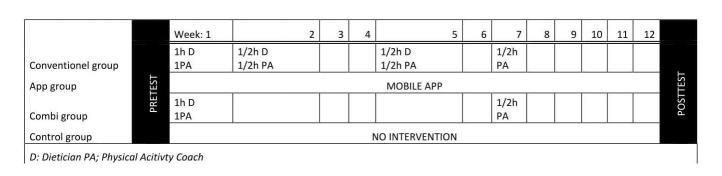
Description of intervention.

Through flyers, social media, and advertisements in local media, overweight and obese adults were invited to participate in a 12-week weight loss intervention. Every person with an interest in the study was invited to attend a general information session about health risks related to overweight, importance of regular physical activity, healthy eating for successful weight loss, and information about this study. After this session, the invitees could sign up for participation in the study. After signing informed consent, the principle investigator allocated the participants in the different groups by means of random number allocation in Excel (Microsoft; see Figure 1). The allocation rate was 1/1/1/1. Participation in this study was free for all participants. All measurements were taken by a blinded assessor. The study took place, including introduction, pre- and postmeasurements, as well as counseling session, at the premises of the Department of Movement Sciences, Physical Activity, Sports and Health Research Group, KU Leuven in Leuven, Belgium. The study was performed from September 2015 to March 2016. All measurements were conducted by registered dieticians and a qualified physical activity coach. Study procedures were approved by the Medical Ethics Committee University Hospital Leuven (registration number S57538). This trial was registered at clinicaltrials.gov, number NCT02595671.

### Interventions

#### Conventional Face-to-Face Weight Loss Program (Conventional Group)

Participants of this group received an individualized diet plan from a registered dietician. Furthermore, each participant received a personalized physical activity plan for 12 weeks from a physical activity coach. In both plans, behavioral change techniques such as self-monitoring, action planning, and relapse prevention were incorporated [9,10]. In the first week, participants had a 1-hour intake with the dietician and a 1-hour intake with the physical activity coach. In the second and fifth week, participants had face-to-face sessions with the dietician (30 min) and physical activity coach (30 min). In the seventh week, participants received an additional session with the physical activity coach. The main advice with regard to nutrition was to reduce their daily energy intake with 500 kcal, a protein intake at 25% of daily energy intake, keep a low glycemic index, and increase intake of fruit and vegetables. The main advice concerning physical activity was to be physically active on at least 5 days a week (preferably all days a week) for at least 30 min at a moderate to high intensity. Participants in this group did not receive access to the mobile weight loss app.

#### A Mobile Weight Loss App (App Group)

**Figure 2:**
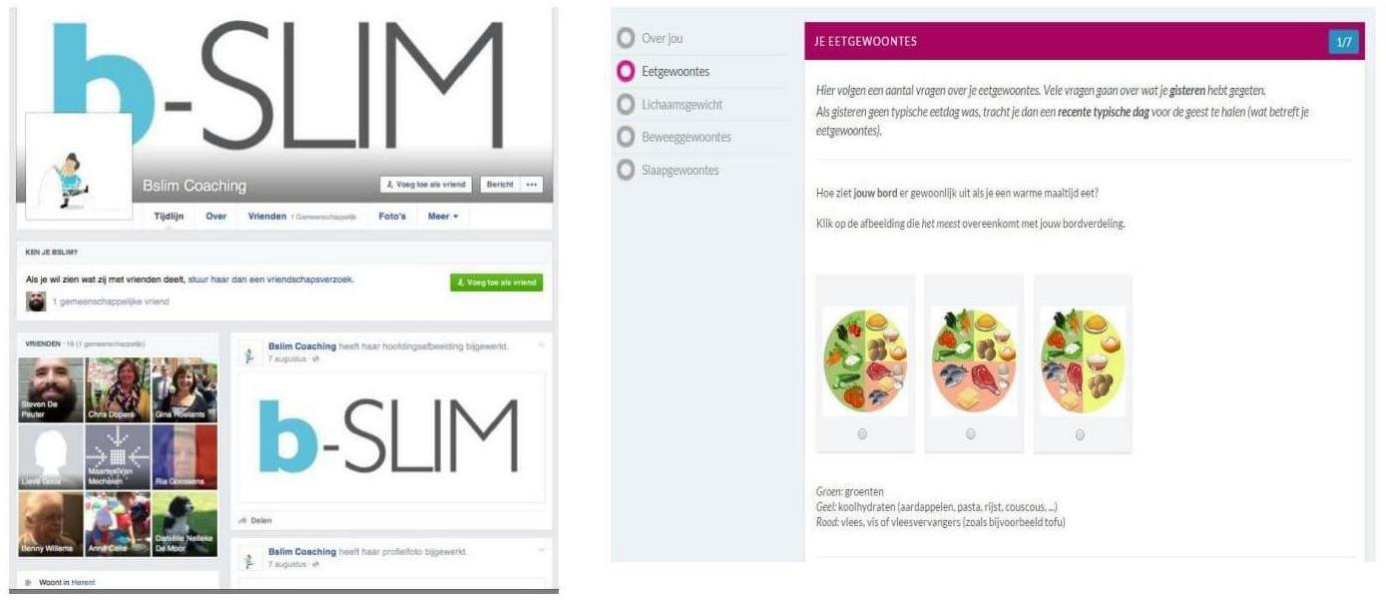
Screenshots of the mobile weight loss app.

Participants in this group received an account to use the digital mobile app. The app consisted of six parts: digital advice for their dietary pattern and physical activity, how to challenge themselves, self-monitoring (step count), library with (scientific) information on nutrition and physical activity but also recipes, a help button for advice, and a link to a Facebook group. The app was available for Android and iPhone operating system (iOS, Apple Inc). The content of the digital advice matched with the conventional advice. See Figure 2 for screenshots of the app.

#### Partial Conventional or Partial Mobile Weight Loss Program (Combi Group)

The subjects of this group received first a 1-hour intake with the dietician and a 1-hour intake with the physical activity coach in the first week. Within these consultations, they received the same information as the conventional treatment group. Additionally, these subjects received an account to use the mobile weight loss app. In the seventh week, participants received an additional face-to-face session with the physical activity coach. Compared with the conventional group, participants received two lesser 30 min counseling sessions with a dietitian and physical activity coach during the intervention.

#### Control Group

Participants were informed that they were on the waiting list for the weight loss program.

### Outcome Measurements

#### Weight Loss

Percentage of participants with at least 5% decrease in baseline weight in kg (5% criterion) [11], BMI (kg/m²) calculated from measured body weight (kg), and body height (m). Weight and height were measured according to the standardized method as described in the World Health Organization, Technical Report Series 854 [12].

#### Cardio-Metabolic Risk Factors

Waist circumference (WC) was measured with an inelastic tape, placed directly on the skin, perpendicularly to the long axis of the body while the subject stood balanced on both feet, with both arms hanging freely [12]. WC of more than 102 cm for men and more than 88 cm for female was taken as abnormal. Blood pressure (BP) was measured with mercury sphygmomanometer in the sitting position after 5 min of rest. Hypertension was defined as BP>130mmHg for systolic or >85mmHg diastolic or on the basis of hypertension treatment.

High density lipoprotein cholesterol (HDL-C) and triglycerides (TG) were determined using a CardioChek Point-of-Care Self-Test device (Cardiochek PA, Polymer Technology Systems Inc., Indianapolis, IN, United States) [13]. TG 150 mg/dl or more and serum HDL-C less than 40 mg/dl for males and less 50 mg/dl for females was considered abnormal. Glucose levels (average of 2 measurements) were measured by the participants themselves in a fasted state by means of the BGStar measurement (Sanofi). Glucose levels of 100 mg/dl or more was considered abnormal.

#### Dietary Pattern

A validated digital Food Frequency Questionnaire (FFQ), developed to estimate the overall dietary pattern, was used to measure dietary changes during the 12-week period [14]. A higher change score on the FFQ means a healthier dietary pattern, including more fruit, vegetable, more water, more fish, less soda and alcoholic drinks, and less meat.

#### Physical Activity Behavior

Physical activity was measured objectively and by means of a self-administrated questionnaire. Objective measurement of physical activity was provided with a tri-axial accelerometer (ActiGraph, model wGT3X-BT, LLC, Pensacola, Florida, United States) [15]. Absolute time spent engaged in moderate (3-5.9 metabolic equivalent of tasks [METS]) and vigorous (≥6METS) intensity activity was calculated (moderate to vigorous physical activity [MVPA]).

The International Physical Activity Questionnaire-Short Form (IPAQ-SF) was used to estimate the amount of self-reported physical activity in the past week [15]. On the basis of these data, participants were categorized into (1) inactive, (2) minimally active, or (3) health enhancing physical activity (HEPA) active [16].

### Statistical Analyses

All results were expressed as mean (standard deviation, SD) and mean difference (SD). Intention-to-treat (ITT) analyses were performed. Differences between groups in the baseline data regarding anthropometric (including BMI), dietary patterns, physical activity, and cardio-metabolic risk factors were analyzed using an analysis of variance (ANOVA) comparison test or chi-square test. BMI, dietary pattern, physical activity, and cardio-metabolic risk factors data were analyzed using a 4 × 2 mixed-model repeated-measures ANOVA with group and time (pre vs post) as factors and gender as covariate. Significant interactions were further analyzed by means of Tukey test *post-hoc* analysis. Statistical significance was conventionally considered as *P* ≤.05. All analyses were performed with Statistical Package for the Social Sciences (SPSS) version 23 (IBM Corp).

The sample size for equivalence studies was calculated based on the 5% criterion for weight loss. On the basis of the results of a recent RCT [17], we estimated the success rates of all intervention to be 37%. Together with a power of 0.80 and a significance level of 5%, this led to a needed sample size of 30 per group.

## Results

### Participants

**Figure 3:**
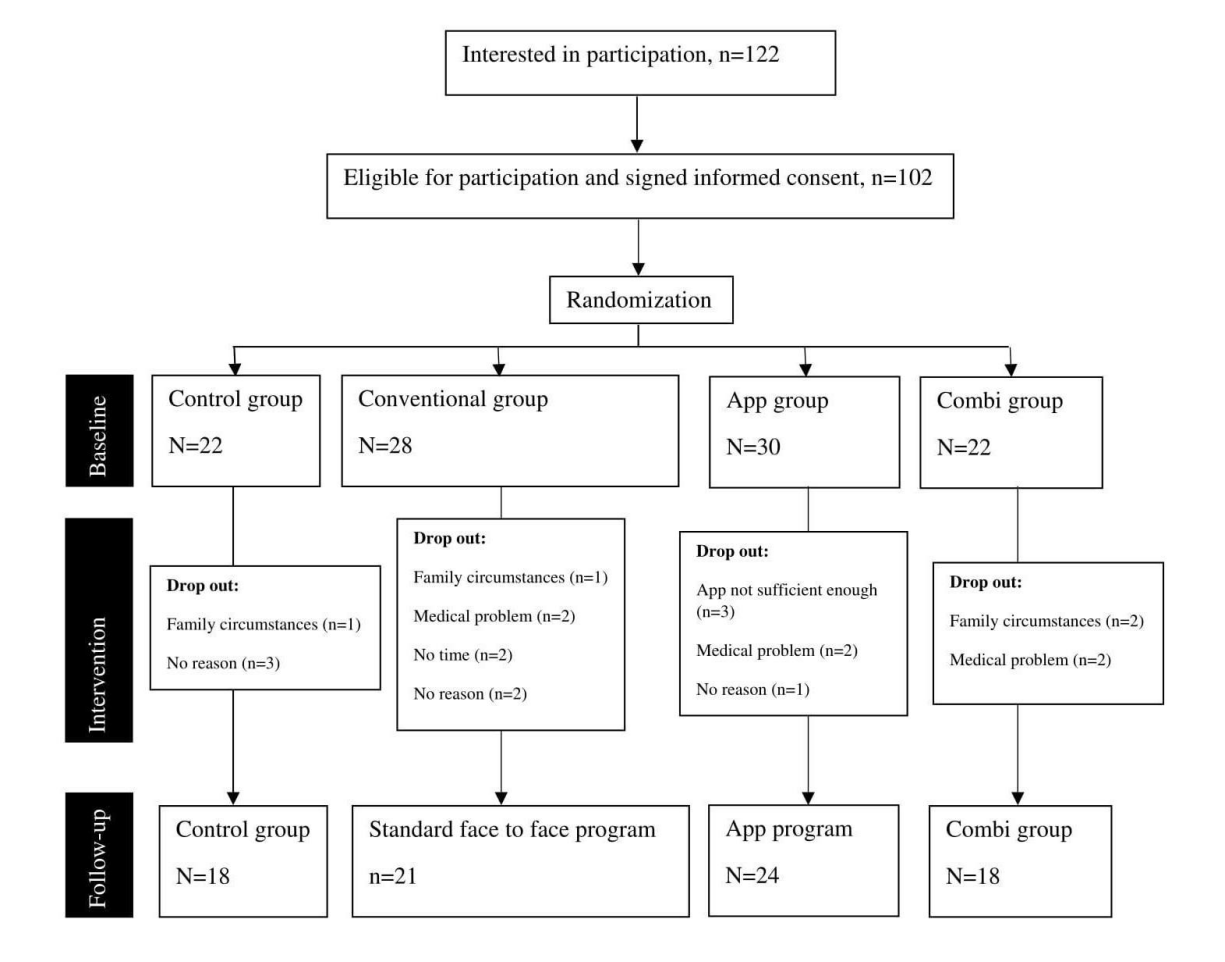
Flowchart of trial.

Of the 122 initially recruited participants, 102 completed the trial (79%; 102/122; see Figure 3). For baseline characteristics see Table 1. There were no baseline differences between the four groups, except for gender (*P*=.02). In the combi group, significant more men were included.

### Weight Loss

**Figure 4:**
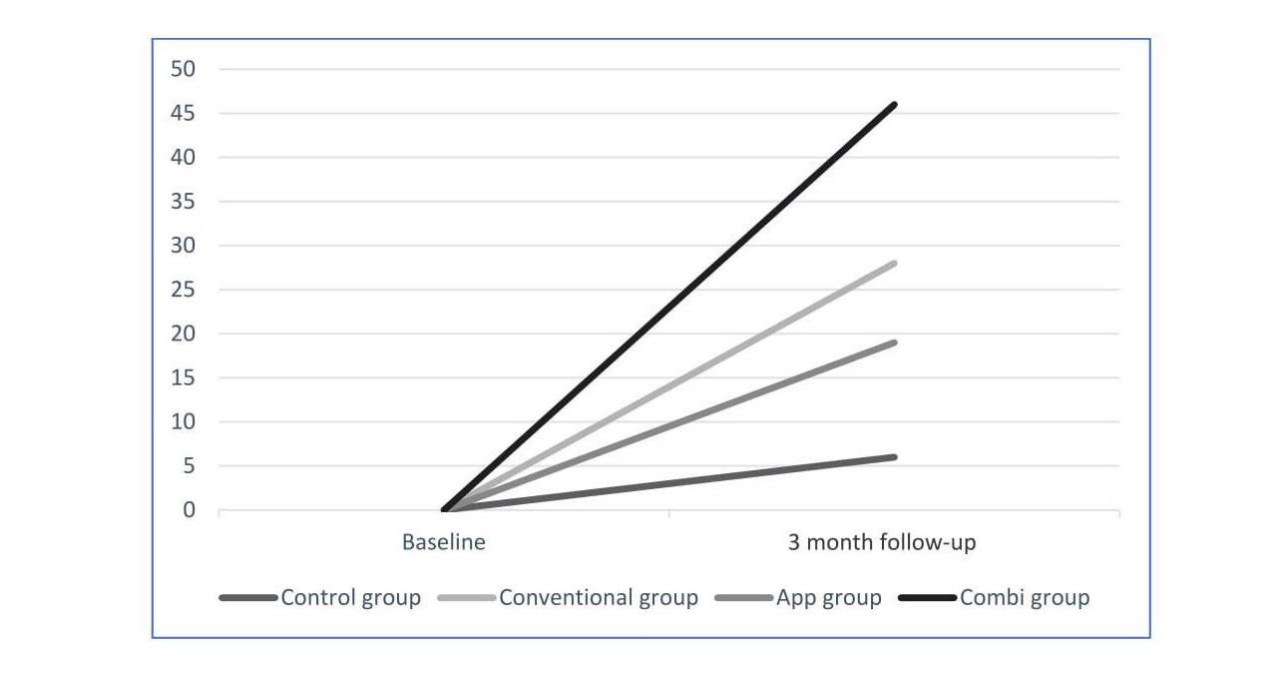
Weight loss (percentage of persons losing 5% of baseline weight).

Significant more participants in all three intervention groups lost at least 5% or more of their weight at baseline compared with the control group (see Figure 4). A trend was found that more participants in the combi group lost 5% or more compared with the app group (19%, *P*=.06). No significant difference was found between the combi group and the conventional group.

A significant time x group effect was found for BMI (*P*=.006), with the control group being significantly different compared with all other intervention groups. No significant decrease was found in the control group. In the conventional group, app group, and combi group, BMI decreased significantly (*P*=.004, *P*=.005, and *P*<.001, respectively; see Table 2).

No significant differences were found between the conventional group and the app group and between the conventional group and the combi group (*P*=.41). However, the combi group had significantly higher decrease in BMI compared with the app group (*P*=.03).

### Cardio-Metabolic Risk Factors

A significant time x group effect was found for cardio-metabolic risk factors (*P*=.05,) with the control group being significant worse compared with all other intervention groups. A nonsignificant increase was found in the control group (*P*=.18). Within the conventional group, app group, and combi group, a decrease in metabolic risk factors was found, but this change was not significant (*P*=.12, *P*=.15, and *P*=.23, respectively; see Table 2).

No significant differences were found between the three intervention groups. However, all intervention groups had significant higher decreases in cardio-metabolic risk factors compared with the control group (all *P*<.05).

### Dietary Pattern

No significant group x time effect was found for dietary pattern (see Table 2). However, a borderline group x time effect was observed for total energy intake (*P*=.05). All groups reduced their total energy intake; however, only significant changes were found within the conventional group (*P*=.001), app group (*P*=.001), and combi group (*P*<.001) and not in the control group (*P*=.22).

### Physical Activity

No significant group x time effects were found for MVPA. Furthermore, no significant changes were found in any of the groups with regard to the percentage of participants that fulfilled the IPAQ minimally active criteria and the HEPA active criteria (see Table 2).

**Table 1:** Baseline characteristics of the participating adults.

Characteristics	Control group (n=18)	Conventional group (n=21)	App group (n=24)	Combi group (n=18)
Age (years), mean (SD^a^)	45 (10.2)	46 (9.2)	44 (12.4)	45 (9.6)
Female (%)	75	84	72	48^b^
Weight (kg), mean (SD)	92 (10.2)	90 (9.1)	90 (10.1)	96 (12.0)

^a^SD: standard deviation.

^b^*P*<.05.

**Table 2:** Changes in body mass index (BMI), metabolic risk factors, physical activity, and dietary pattern.

Factors		Control group (n=22)		Conventional group (n=28)		App group (n=30)		Combi group (n=22)	
		Pre, mean difference (SD^a^) or n (%)	Post, mean difference (SD)	Pre, mean difference (SD) or n (%)	Post, mean difference (SD)	Pre, mean difference (SD) or n (%)	Post, mean difference (SD)	Pre, mean difference (SD) or n (%)	Post, mean difference (SD)
BMI^b^		32 (2.0)	0.1 (1.0)	32 (2.0)	−1.0 (1.3)	32 (2.1)	−0.7 (1.0)	32 (2.2)	−1.3 (1.2)
Metabolic risk		2.9 (1.2)	0.5 (1.4)	3.0 (1.0)	−0.6 (1.4)	3.2 (1.3)	−0.5 (1.5)	2.9 (1.0)	−0.3 (1.1)
**Physical activity**									
	Category 2	17 (59%)	0 (0.7)	16 (64%)	0.11 (0.6)	14 (58%)	0.05 (0.6)	20 (71%)	0.04 (0.6)
	HEPA^c^	6 (21%)	−0.06 (0.4)	7 (28%)	0.0 (0.6)	3 (11%)	−0.04 (0.5)	7 (25%)	−0.08 (0.5)
	MVPA^d^ (min)	324 (89)	−1.7 (63.7)	314 (82)	11.8 (61.4)	333 (82)	3.6 (72.8)	348 (90)	−33.5 (39.8)
Overall score nutrition pattern		69.5 (13.1)	3.0 (6.8)	69.7 (11.5)	11.,2 (13.7)	71.5 (12.6)	8.1 (13.8)	70.0 (14.9)	8.7 (12.6)
Energy intake (kcal)		1534.2 (548.3)	−115.1 (381.8)	1453.3 (413.6)	−392.7 (302.9)	1489.5 (414.9)	−192.2 (247.4)	1456.5 (397.6)	−287.3 (277.3)

^a^SD: standard deviation.

^b^BMI: body mass index.

^c^HEPA: health enhancing physical activity.

^d^MVPA: moderate to vigorous physical activity.

## Discussion

### Principal Findings

This study evaluated whether conventional weight loss programs could be (partially) completed with an mHealth app. The results of our study show that when replacing a part of the conventional program by an mHealth app does not affect the effectiveness of the program. Although an mHealth app as a single intervention also showed positive results on BMI and weight reduction, this change was smaller compared with the conventional and combi group.

### Comparison With Prior Work

Our results with regard to BMI and weight reduction are in line with previous studies [18-20]. Most studies show that an mHealth app leads to weight reduction. However, the extent of weight loss reduction varies per study. In our study, weight loss was relatively high within the app group. This could be explained by the inclusion of behavioral components such as self-monitoring, avatar possibility (adjustable image of themselves withouta number of kilograms), action planning, and relapse prevention in the current app, which enables the participants to change their health behavior and maintain this behavioral change during the whole 12-week intervention period. This approach is in contrast with many apps that had not (or not sufficiently) incorporated a behavioral component [5,6].

In our study, metabolic risk factors decreased in our intervention groups. These results are in line with other studies, showing that a combination of a calorie intake reduction combined with physical activity reduces metabolic risk factors [21,22]. However, within the control group, metabolic risk factors increased while they also decreased their energy intake. This could indicate that there is a threshold for the level of energy intake reduction to have an effect on metabolic risk factors.

All groups in our study reduced their energy intake during the trial period. The conventional group and combi group showed highest decrease. Interestingly, the reduced energy intake was accompanied with an overall improvement of their dietary quality. This could be the added value of a health professional. They monitor the patient and provide individualized advice and personalized solutions to certain person specific problems. In an mHealth app, such a personalized approach is not possible to such an extent. Furthermore, this interaction with a health professional might be of high importance for long-term maintenance of the results. Future long-term studies should further evaluate the most effective combination of a health professional and an mHealth app.

Most previous studies showed that conventional weight loss programs, as well as mobile weight loss programs, have a positive effect on the level of physical activity [23]. In our study, no significant improvement was found. This could be explained by the fact that a high percentage of our sample population was already physically active at baseline. More than 60% of our participants reported that they were already active on at least 5 days a week, 30 min on a moderate intensity level or 3 times a week on a high intensity level. The main advice concerning physical activity in our study was to be physically active on at least 5 days a week (preferably all days a week) for at least 30 min at a moderate to high intensity. If participants felt that they were already active enough, it is possible that the goal setting approach to increase their daily level of physical activity could have been marginalized or ignored. Although we have to interpret these results with caution as they are based on a self-reported questionnaire, it could explain why our participants did not significantly increase their physical activity levels. Nonetheless, adults’ activity levels have been shown to vary depending on season of measurement, with lower levels of activity in the winter versus spring or summer months [24]. This study was conducted from September 2015 to March 2016, with most participants enrolled by midautumn and followed up through winter. Despite this expected decrease in adult’s physical activity levels because of seasonal variation, participants in this study were successful in maintaining their activity level during winter months. This was also the case in the control condition. This may be because regular measurement can encourage participants to reflect on their physical activity [25].

The amount of sessions in our study might be different compared with other similar studies. In our conventional face-to-face weight loss program, the number of sessions with a dietician was based on the standard of care and the number of sessions that are financially reimbursed through the Belgian social security system. Unfortunately, there is currently no financial reimbursement for the sessions with a physical activity coach in Belgium. Therefore, the content and number of sessions in the conventional face-to-face weight loss program were based on their hypothesized effectiveness from previous research among Belgian adults [26]. Compared with the conventional face-to-face weight loss program, the number of face-to-face contacts with a dietician and physical activity coach were reduced in the combi group intervention (partial conventional or partial mobile weight loss program) to offer the participants a feasible blended intervention condition. Furthermore, both the conventional group and the combi group were offered an additional session of 30 min with a professional physical coach in week 7. In both the conventional face-to-face weight loss program, as well as in the combi group, this additional session with a professional physical activity coach halfway through the intervention period (week 7) specifically targeted self-efficacy by using self-regulation techniques such as action planning and relapse prevention techniques (ie, barriers and solutions) that can be applied to all behavior changes (physical activity as well as food intake).

### Limitations

Our study has a few limitations that should be kept in mind. The first limitation of this study is the sample size. Although 102 participants started the trial, some participants dropped out during the trial (n=21). However, when using the data of our combi group, a sample size of 15 would have been required. Furthermore, to see whether the data of these dropouts affected our results, a per-protocol and an ITT analyses was performed, which showed no differences on the main outcomes. A second limitation is the use of the accelerometer on the wrist. The data with regard to physical activity levels in our study were relatively high. Previous studies have already shown that the data from the accelerometer depends on the location of measurement on the body [27]. Additionally, the IPAQ has been shown to overestimate the amount of physical activity [28]. Unfortunately, a more accurate device or questionnaire is currently not available. A third limitation is the self-reported glucose measurement. They were instructed to take two consecutive measurements in a fasted state. Whether participants followed these instructions remains unclear. A forth limitation is the duration of our intervention of 3 months. The duration and frequency of the intervention was based on the weight loss programs adults in Belgium would currently receive. In addition, the aim of this study was not to evaluate the difference in long-term effects. Our aim was to initially evaluate whether current conventional care could be replaced by a mobile app. A fifth limitation is the way weight change was measured. In our study, the 5% weight loss criterion was used instead of actual weight lost because the first 5% of weight loss offers the greatest health benefits. Although weight loss expressed in kg would be interesting, the most important outcome is whether people actually achieve a level where positive effects on health occur.

### Conclusions

In conclusion, our study showed that a conventional weight loss program could partially be completed with an mHealth app without affecting the effectiveness. Such combined approach could support health professionals and reduce their workload. Further ways of combining conventional weight loss programs with mHealth apps should be further explored. Whether such combined programs are also cost-effective should be further investigated. Furthermore, long-term studies should evaluate whether the effects of a combined program can be maintained over a long time period.

## Appendix

Multimedia Appendix 1 CONSORT‐EHEALTH checklist (V 1.6.1).

## Abbreviations

ANOVA: analysis of variance
BMI: body mass index
BP: blood pressure
FFQ: Food Frequency Questionnaire
HDL-C: high density lipoprotein cholesterol
HEPA: health enhancing physical activity
IPAQ-SF: International Physical Activity Questionnaire-Short Form
ITT: intention-to-treat
METS: metabolic equivalent of tasks
mHealth: mobile health
MVPA: moderate to vigorous physical activity
RCT: randomized controlled trial
SD: standard deviation
TG: triglycerides
WC: waist circumference
