# Acknowledgement Correction: Face-to-Face Versus Mobile Versus Blended Weight Loss Program: Randomized Clinical Trial

**DOI:** 10.2196/10159

**Published:** 2018-03-15

**Authors:** Emalie Hurkmans, Christophe Matthys, An Bogaerts, Leonie Scheys, Karlien Devloo, Jan Seghers

**Affiliations:** ^1^ Department of Movement Sciences University of Leuven Leuven Belgium; ^2^ Department of Social Affairs and Health Ecorys Rotterdam Netherlands; ^3^ Department of Endocrinology University Hospitals Leuven Leuven Belgium; ^4^ Department of Chronic Diseases, Metabolism and Ageing University of Leuven Leuven Belgium; ^5^ Department of Clinical and Experimental Endocrinology University of Leuven Leuven Belgium; ^6^ Faculty of Movement and Rehabilitation Sciences University of Leuven= Leuven Belgium

The authors of “Face-to-Face Versus Mobile Versus Blended Weight Loss Program: Randomized Clinical Trial” (JMIR mHealth uHealth 2018;6(1):e14) would like to change the Acknowledgments section of their paper to the following: 

This project is partially funded and realized in collaboration with imec, Belgium. BrandNewHealth developed the weight loss app.

The corrected article will appear in the online version of the paper on the JMIR website on March 15, 2018, together with the publication of this correction notice. Because this was made after submission to PubMed or Pubmed Central and other full-text repositories, the corrected article also has been re-submitted to those repositories.

